# Evolutionary characteristics of the *GAPDH* gene family and functional characterization of *TaGAPDH5 *in wheat

**DOI:** 10.1186/s12870-026-09787-x

**Published:** 2026-09-24

**Authors:** Huaqing Li, Yang Liu, Pengxia Guo, Yesong Tian, Yang Yu, Teng Li, Yulong Song, Weiguo Hu, Donghong Min

**Affiliations:** 1 Hainan Institute of Northwest A&F University, Sanya, Hainan 572025 China; 2 College of Agronomy, State Key Laboratory of Crop Stress Biology for Arid Areas, Northwest A&F University, Yangling, 712100 Shaanxi China; 3 Institute of Wheat, Henan Academy of Agricultural Sciences, Zhengzhou, 450002 Henan China

**Keywords:** *Triticum aestivum*, *GAPDH* gene family, Evolutionary analysis, Drought stress, *TaGAPDH5*

## Abstract

**Background:**

Glyceraldehyde-3-phosphate dehydrogenase (GAPDH) is a key glycolytic enzyme that also plays important roles in plant responses to abiotic stresses. However, the evolutionary relationships of the *GAPDH* genes among wheat species with different ploidy levels remain poorly understood, and the key *TaGAPDH* members involved in the drought response of wheat have yet to be identified.

**Results:**

In this study, we identified 5, 6, 13, and 19 GAPDH proteins in *Triticum urartu*, *Aegilops tauschii*, *Triticum turgidum*, and *Triticum aestivum* with different ploidy levels. All identified GAPDH proteins harbored the conserved Gp_dh_N and Gp_dh_C domains and were phylogenetically assigned to four subfamilies: GAPA, GAPB, GAPC, and GAPCp. Gene structure and conserved motif analyses indicated overall conservation of the *GAPDH* family but clear divergence among subfamilies, with Motif 13 emerging as a characteristic motif unique to GAPB. *Cis*-element prediction revealed that the promoters of *TaGAPDH* genes contain numerous putative *cis*-acting elements associated with responses to drought and light. Transcriptomic profiling further verified that *TaGAPDH* genes exhibited tissue-specific expression patterns and were actively responsive to drought stress. Expression trend correlation analysis of drought-responsive transcriptomic data suggested a potential regulatory association between TaMYB-7D and *TaGAPDH5*. Consistently, transient dual-luciferase assays showed that TaMYB-7D activates the *TaGAPDH5* promoter. Under drought stress, *TaGAPDH5* overexpressing *Arabidopsis thaliana* lines displayed less intense DAB and NBT staining, higher soluble sugar contents, reduced wilting, and fewer dead leaves than wild type plants.

**Conclusions:**

Collectively, this study characterized *GAPDH* gene family members in wheat species of different ploidy levels and identified *TaGAPDH5* as a potential drought tolerance candidate, providing a basis for further functional validation and possible application in drought tolerance breeding.

**Supplementary Information:**

The online version contains supplementary material available at https://doi.org/10.1186/s12870-026-09787-x.

## Background

Wheat (*Triticum aestivum*) is a vital food crop that sustains nearly half of the global population [1, 2]. As its primary production areas are concentrated in arid and semi-arid regions, drought is one of the major abiotic stressors limiting yield. This threat is increasingly exacerbated by global warming and population growth [3–5]. Consequently, identifying and deploying drought resistance genes is essential for stabilizing wheat production. Drought tolerance in wheat is a complex quantitative trait regulated by intricate genetic networks spanning signal transduction [6], transcriptional regulation [7], and physiological and metabolic pathways [8]. Nevertheless, many genes involved in drought resistance remain unidentified, and the molecular mechanisms governing their roles in drought adaptation are still incompletely understood. Enhancing yield stability under drought stress thus remains a critical priority for wheat breeding programs [9].

Plant drought responses are governed by the functional enzymes and transcription factors (TFs). Functional enzymes drive metabolic reprogramming and physiological adaptation. Specifically, proline dehydrogenase (PRODH) facilitates osmolyte synthesis, while superoxide dismutase (SOD), peroxidase (POD), and catalase (CAT) maintain reactive oxygen species (ROS) homeostasis. Additionally, enzymes such as GAPDH mediate glycolysis and energy metabolism, both of which are essential for drought adaptation [10, 11]. As upstream regulatory hubs, TFs including NAM, ATAF1/2, and CUC2 (NAC), the MYB transcription factor (MYB), basic Leucine Zipper (bZIP), and the nuclear factor Y (NF-Y) recognize promoter *cis*-acting elements to trigger transcriptional cascades. These factors regulate processes such as stomatal movement, osmolyte accumulation, and senescence to enhance drought tolerance [12–16]. These regulators operate interdependently, TFs activate the expression of enzyme encoding genes, while enzymatic metabolites can feedback regulate TF activity, forming complex cooperative circuits [17, 18]. Consequently, systematically dissecting these collaborative modes is vital for unveiling the molecular mechanisms of drought tolerance and identifying genetic resources for breeding drought resistant varieties.

GAPDH is a fundamental glycolytic enzyme that catalyzes the oxidation of glyceraldehyde-3-phosphate to 1,3-bisphosphoglycerate. Although traditionally utilized as a stable reference gene, recent evidence indicates that the *GAPDH* gene expression is significantly influenced by environmental stress, rendering it unsuitable as a conserved housekeeping gene in many contexts [19–22]. Beyond its primary metabolic role, *GAPDH* possesses diverse moonlighting functions that regulate plant growth, development, and stress adaptation. Its biological activity is further modulated by post translational modifications, including S-nitrosylation and phosphorylation [23–26]. Additionally, GAPDH acts as a pivotal regulatory node in signaling through various molecular interactions and upstream transcriptional control. For example, TaWRKY40 enhances drought tolerance in wheat by positively regulating *TaGAPC1* expression [27]. Although the *GAPDH* gene family has been systematically characterized in several plant species, including watermelon, pepper, rapeseed, and poplar, where its members exhibit functional diversification in salt-stress responses, antiviral defense, and hormone signaling [22, 28–30]. Nevertheless, the wheat *GAPDH* gene family remains incompletely characterized, particularly with respect to promoter *cis*-regulatory architecture, drought-responsive expression dynamics, protein interaction networks, and the functional contributions of individual members to drought tolerance. Comparative genomic and evolutionary analyses across diploid, tetraploid, and hexaploid wheat are therefore needed to resolve the evolutionary history and functional diversification of *GAPDH* genes during polyploidization and drought adaptation. These efforts will facilitate the identification and functional validation of key *GAPDH* candidates for the genetic improvement of drought-resilient wheat.

In this study, we systematically identified *GAPDH* genes in *Triticum aestivum* and its diploid and tetraploid progenitors and investigated the evolutionary dynamics of this family during wheat polyploidization through phylogenetic reconstruction, chromosomal mapping, synteny analysis, and duplication-event analysis. Integrative analyses of gene structure, conserved domains, promoter *cis*-regulatory elements, protein-protein interaction networks, and expression patterns across tissues and under drought stress revealed structural divergence and potential functional differentiation among *GAPDH* subfamilies. Members of the GAPA and GAPB subfamilies were potentially associated with drought responses, with *TaGAPDH5* identified as a priority candidate for further functional investigation. Dual-luciferase assays showed that TaMYB-7D activated the *TaGAPDH5* promoter, suggesting that it may participate in the transcriptional regulation of *TaGAPDH5*. Moreover, heterologous expression of *TaGAPDH5* enhanced drought tolerance in *Arabidopsis thaliana*, supporting its potential role in drought-stress adaptation. Collectively, these findings provide an evolutionary and functional framework for prioritizing drought-responsive *GAPDH* genes and offer candidate genetic resources for molecular breeding of drought-resilient wheat.

## Materials and methods

### Genomic data retrieval and identification of the *GAPDH* gene family

Genome sequences, protein sequences, and annotation files of *Triticum aestivum*, *Triticum urartu*, *Triticum_turgidum*, *Aegilops tauschii*, *Oryza sativa*, *Zea mays*, and *Arabidopsis thaliana* were retrieved from the Ensembl Plants database. The hidden Markov model (HMM) of the GAPDH domain was obtained from the Pfam database. Candidate GAPDH sequences were screened based on the conserved Gp_dh_N (PF00044) and Gp_dh_C (PF02800) domains, and the longest coding transcript of each gene was retained [31]. Furthermore, the GAPDH protein sequences of *Arabidopsis thaliana* and *Oryza sativa* were used as probes to preliminarily screen wheat GAPDH candidates via the built-in BLAST tool in TBtools-II [32–34]. Conserved domains were further verified using the SMART, NCBI CDD, and InterPro databases to validate the identities of the identified the wheat GAPDH family members [35–37]. Protein physicochemical properties were determined using the ExPASy online server [38].

### Phylogenetic analysis, synteny, and gene duplication

GAPDH protein sequences from *Triticum aestivum*, *Triticum urartu*, *Triticum_turgidum*, *Aegilops tauschii*, *Oryza sativa*, *Zea mays*, and *Arabidopsis thaliana* were aligned. A phylogenetic tree was constructed via the neighbor-joining method in MEGA11 with 1000 bootstrap replicates, and the resulting tree was further optimized using Evolview_v2 [39, 40]. To explore the evolutionary relationships of *GAPDH* genes across different species, interspecific collinearity analysis and visualization were performed using the MCScanX program embedded in TBtools-II. The duplication patterns of wheat *GAPDH* genes were identified using the default parameters of MCScanX and the results were visualized with Circos_v0.69.9 [41, 42]. Additionally, the nonsynonymous and synonymous substitution rates of homologous gene pairs were calculated in TBtools-II to assess the selective pressure acting on the wheat *GAPDH *gene family [34].

### Chromosomal localization, gene structure, and conserved motifs

The chromosomal locations, sequence lengths, and structural features of wheat *GAPDH* gene family members were retrieved from the Ensembl Plants database. Chromosomal distribution maps were generated using MG2C_v2.1 [43]. Conserved protein motifs were predicted via MEME [44], and the resulting motif patterns and gene structures were integrated and visualized using Evolview_v2. Multiple sequence alignment was performed using DNAMAN v9.0 (Lynnon Biosoft, San Ramon, CA, USA).

### Promoter analysis of *TaGAPDH* genes

The 2000 bp upstream sequences of the start codon for each *TaGAPDH* genes were extracted, and *cis*-acting regulatory elements were subsequently identified and annotated using the PlantCARE database [45].

### GO enrichment analysis and protein-protein interaction network prediction

Gene Ontology (GO) annotations for *TaGAPDH* genes were retrieved via the Ensembl Plants BioMart tool to conduct functional enrichment analysis. Protein-protein interaction (PPI) networks were predicted by submitting TaGAPDH protein sequences to the STRING database, using *Arabidopsis thaliana* as the reference species with a high confidence interaction score threshold (≥ 0.7) and a limit of 10 interactors per protein [46]. Both GO enrichment results and the PPI network were visualized using Cytoscape_v3.10.3 [47].

### Tissue-specific expression and drought stress response analysis of *TaGAPDH* genes

Public transcriptomic datasets covering various tissues, developmental stages, and responses to natural drought and PEG6000 simulated drought stress were retrieved from the WheatOmics database [48]. Expression levels of *TaGAPDH* genes were extracted, and their expression profiles were visualized using heatmaps generated with TBtools-II to analyze spatiotemporal and stress responsive patterns.

### Prediction of putative upstream transcription factors and construction of a regulatory network for *TaGAPDH* genes

Putative upstream transcription factors (TFs) of wheat *GAPDH* family members were predicted using the wheat gene regulatory network (wGRN) platform. Candidate regulatory relationships between TFs and wheat *GAPDH* family genes were initially screened based on a fold enrichment of ≥2 and an FDR of <0.01. Drought-stress transcriptome data were subsequently integrated to assess the consistency of expression trends between candidate TFs and *GAPDH* genes. Putative TF–target relationships exhibiting high concordance in expression trends were retained for downstream network construction using a correlation-based filtering approach [49]. Specifically, candidate interactions with *r* ≥ 0.95 and *P* < 0.05 were retained. The resulting putative TF-*GAPDH* regulatory network was constructed and visualized using Cytoscape_v3.10.3.

### Plant materials and growth conditions

Genomic DNA and total RNA were extracted from the wheat cultivar Chinese Spring (CS) for promoter amplification and gene cloning, with the RNA subsequently reverse transcribed into cDNA. CS plants were grown in a controlled incubator at 18–22 °C under a 16 h light/8 h dark photoperiod. 2-week-old leaves were harvested for nucleic acid extraction. The wheat cultivar Kenong 199 (KN199) was used for tissue-specific expression profiling and time course expression analysis under PEG6000 induced osmotic stress. For tissue-specific analysis, roots, stems, leaves, spikes, and grains were collected from healthy field grown plants, immediately snap frozen in liquid nitrogen, and stored at -80 ℃ until use. To simulate drought stress, uniform seeds were surface-sterilized with 2% H₂O₂ and germinated in the dark. Germinated seedlings were transferred to a hydroponic system and grown at 20 ± 2 ℃ under a 16 h light/8 h dark photoperiod. 10-day-old seedlings were subjected to osmotic stress using 20% (w/v) PEG6000, and leaf samples were harvested at 0, 3, 6, 12, 24, and 48 h post-treatment.

All *Arabidopsis thaliana* plants used in this study were in the Columbia-0 (Col-0) genetic background. Col-0 plants were used as the wild-type (WT) control and as the recipient genotype for generating independent *TaGAPDH5* overexpression (OE) lines, which were subsequently subjected to functional analyses. Seeds were germinated on half-strength Murashige and Skoog (MS) medium containing 0.8% (w/v) agar and 1% (w/v) sucrose. After stratification at 4 ℃ in the dark for 3 days, the plates were transferred to a growth chamber at 20 ± 2 ℃ (day/light) under a 16 h light/ 8 h dark photoperiod, with an illuminance of 12,000 lx. After 10 days of growth, robust seedlings were transplanted into soil for further phenotypic and functional analyses. *Nicotiana benthamiana* plants were cultivated in a growth chamber at 22 ± 2 ℃ under a 16 h light/8 h dark photoperiod, with an illuminance of 12,000 lx.

### Dual-luciferase reporter assay

To verify the transcriptional regulatory relationship between TaMYB-7D (TraesCS7D02G032700.1) and *TaGAPDH5*, a dual-luciferase reporter assay was conducted. The coding sequence of TaMYB-7D was inserted into the effector vector to generate the 35 S::*TaMYB-7D* effector construct. The promoter fragment of *TaGAPDH5* was amplified and cloned into a reporter vector to obtain the recombinant reporter construct *proTaGAPDH5*::LUC. The recombinant constructs were co-infiltrated into *Nicotiana benthamiana* leaves, with empty vectors co-infiltrated as negative controls and combinations of empty and recombinant vectors used as additional controls. A PlantView100 imaging system was applied to detect the fluorescence intensity of *Nicotiana benthamiana* leaves, and the regulatory interaction was determined according to variations in fluorescence signals.

### TaGAPDH5 protein structure prediction

The secondary structure of TaGAPDH5 was predicted using the SOPMA tool [50]. Its three dimensional (3D) structural model was subsequently constructed using the SWISS-MODEL platform [51].

### Total RNA isolation and reverse transcription quantitative PCR (RT-qPCR) analysis

Total RNA was extracted using the Vazyme RNA extraction kit and reverse transcribed with the FastQuant RT Super Mix kit according to the manufacturer’s protocols. Gene specific primers were designed using DNAMAN and validated via NCBI Primer-BLAST (Table S1). RT-qPCR was conducted with three biological replicates, and relative expression levels were calculated using the 2^−ΔΔCt^ method. *TaEF1a* served as internal controls for wheat [52].

### Heterologous functional validation of *TaGAPDH5* gene

To characterize the biological function of *TaGAPDH5*, a heterologous overexpression assay was performed in *Arabidopsis thaliana*. The full-length coding sequence of *TaGAPDH5* was amplified and inserted into the pBI121 vector under the control of the CaMV 35 S promoter to generate the overexpression construct. The recombinant plasmid was transformed into *Agrobacterium tumefaciens* strain GV3101 and subsequently introduced into WT *Arabidopsis thaliana* via the floral dip method. Stable T3 homozygous transgenic lines were screened for subsequent phenotypic identification. Drought tolerance of the transgenic lines was evaluated by observing their phenotypes under drought stress. All primers used for vector construction are listed in Table S1.

### Measurement of physiological indicators

Histochemical detection of H_2_O_2_ in *Arabidopsis thaliana* leaves was performed using 3,3’ -diaminobenzidine (DAB; purity ≥ 98.0%; catalog no. D6231D, Biotopped). Before staining, a 1 mg/mL DAB working solution was prepared in 50 mM phosphate buffer (pH 5.5). Leaf samples were infiltrated with the DAB solution and incubated in the dark at 28 ℃ for 6–8 h, after which staining patterns were observed and recorded.

Reactive oxygen species (ROS) levels in leaves were detected using nitroblue tetrazolium (NBT) staining, and soluble sugar content was determined with a commercial assay kit. Both assays were performed according to the manufacturer’ s instructions (Beijing Solarbio Science & Technology Co., Ltd., China). NBT staining was used to visualize ROS accumulation and evaluate oxidative stress damage in plant tissues.

### Statistical analysis

All experiments were performed with three independent biological replicates, and all data are presented as the mean ± standard deviation (SD). One-way analysis of variance (ANOVA) was conducted using SPSS 27.0, and Tukey’s multiple comparison test was applied to determine significant differences. Graphical visualization was performed using GraphPad Prism 10. Figures were assembled and annotated using Adobe Illustrator 2025 (Adobe Inc., San Jose, CA, USA).

## Results

### Genome-wide identification and evolutionary analysis of the *GAPDH* gene family

To characterize the evolution of the GAPDH family during wheat polyploidization, we identified 5, 6, 13, and 19 GAPDH proteins in *Triticum urartu* (2n = 2x = 14, AA), *Aegilops tauschii* (2n = 2x = 14, DD), *Triticum turgidum* (2n = 4x = 28, AABB), *Triticum aestivum* (2n = 6x = 42, AABBDD), respectively (Table S2). All identified members contained the characteristic Gp_dh_N and Gp_dh_C conserved domains. Gene distribution analysis revealed that *GAPDH* copy numbers generally reflect genomic composition. While the gene count in tetraploid wheat matches the combined total in the hexaploid A and B subgenomes, *Triticum urartu* possesses fewer genes than the hexaploid A subgenome, suggesting gene expansion within the A subgenome during polyploidization. Overall, the wheat *GAPDH* gene family did not follow a simple linear expansion pattern, its evolution was likely shaped by differential subgenome retention, the emergence of new copies, providing insights into the evolutionary dynamics of wheat polyploidization.

**Fig. 1:**
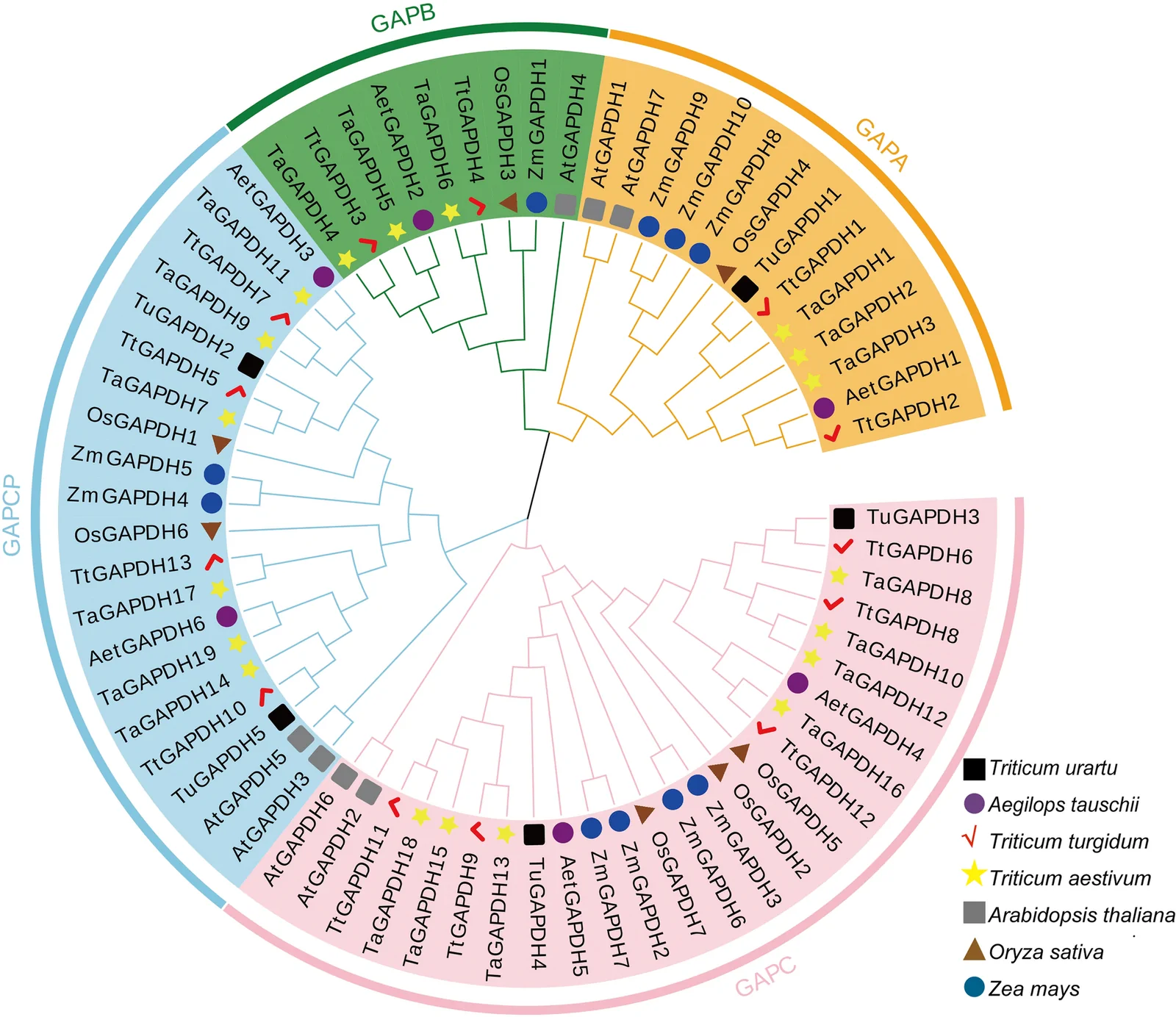
Phylogenetic relationships of GAPDH proteins across diverse species. The neighbor-joining tree illustrates the evolutionary classification of GAPDH family members into distinct subfamilies, represented by different background colors and labels on the outer ring. Markers of various shapes and colors distinguish each species

To further elucidate evolutionary relationships, *GAPDH* genes were identified in *Arabidopsis thaliana* (7), *Oryza sativa* (7), and *Zea mays* (10) (Table S2). A neighbor-joining (NJ) phylogenetic tree was constructed using 67 protein sequences from the seven species, classifying them into four subfamilies: GAPA (13 members), GAPB (9 members), GAPC (25 members), and GAPCP (20 members) (Fig. 1). The variation in member numbers across subfamilies suggests differential expansion and retention during evolution, which may have led to functional divergence. Phylogenetic clustering showed that wheat GAPDH proteins grouped by sequence similarity rather than species ploidy level, indicating that subfamily differentiation preceded the divergence of diploid wheat ancestors.

### Collinearity analysis

#### Collinearity analysis among wheat species with different ploidy levels

**Fig. 2:**
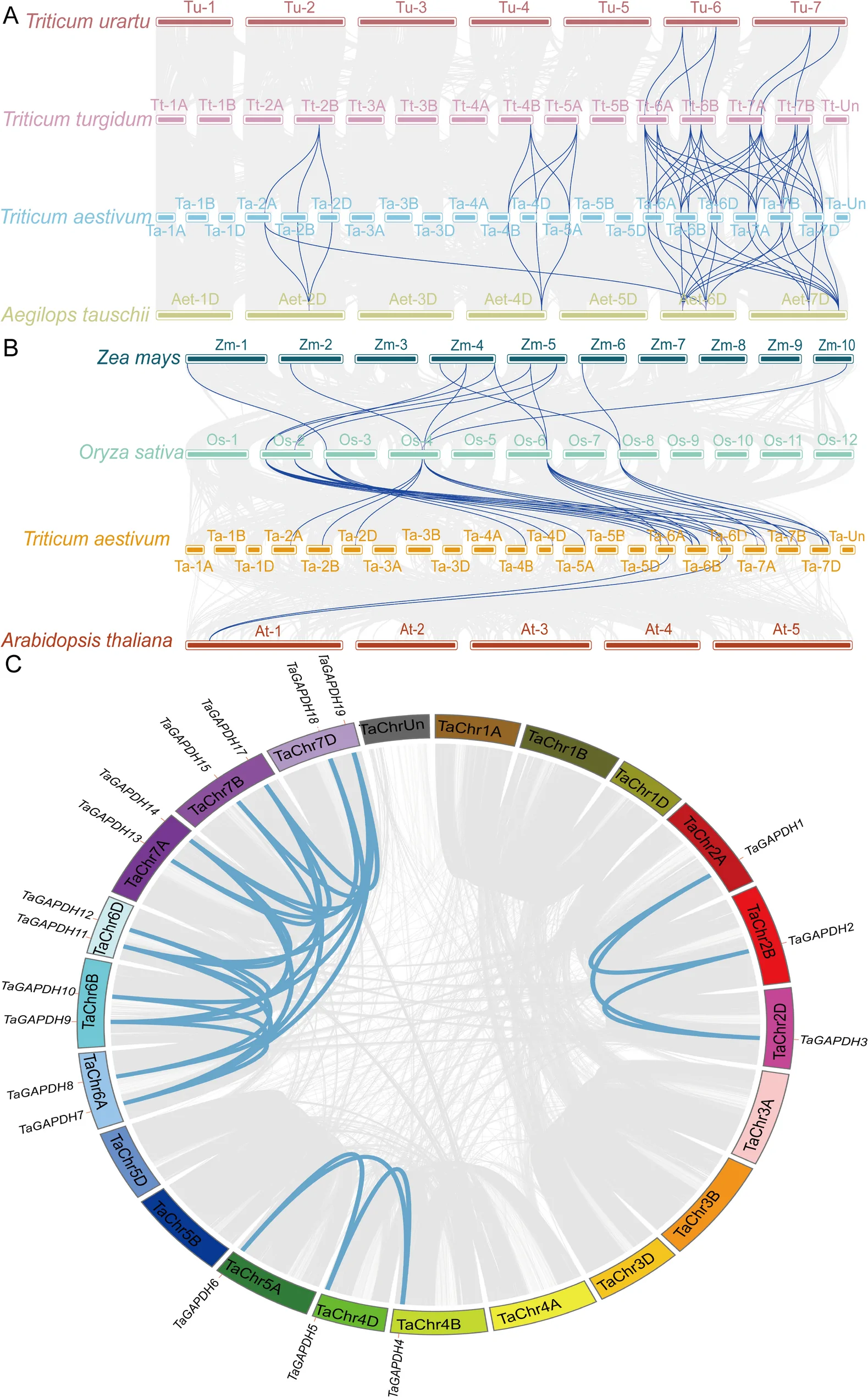
Collinearity and homology analyses of the *GAPDH* gene family. **A** Interspecific synteny analysis of *GAPDH* genes among diploid *Triticum urartu* and *Aegilops tauschii*, tetraploid *Triticum turgidum*, and hexaploid *Triticum aestivum*. **B** Interspecific synteny analysis between wheat and other species (*Oryza sativa*, *Zea mays*, and *Arabidopsis thaliana*). Blue curves represent collinear gene pairs, while grey lines indicate background syntenic blocks. **C** Intraspecific collinearity analysis of the wheat *GAPDH* gene family. Light blue curves represent collinear gene pairs, grey lines denote genomic syntenic blocks, and colored blocks represent chromosomes

To investigate the impact of polyploidization on the structural evolution of the wheat *GAPDH* gene family, we performed comparative collinearity analysis across wheat species with different ploidy levels (Fig. 2A and Fig. S1A). Most *GAPDH* genes were located within conserved syntenic blocks. Diploid and tetraploid species predominantly exhibited single copy colinear gene pairs, whereas hexaploid wheat displayed a 1:3 copy correspondence pattern in homologous regions. This reflects how gene dosage effects drove copy number expansion during subgenome duplication. While most *GAPDH* loci were conserved during polyploidization, some gene loss and uneven collinearity distribution were observed. Specifically, only chromosomes 6 and 7 of *Triticum urartu* shared syntenic segments with the hexaploid A subgenome, whereas multiple chromosomes in *Aegilops tauschii* formed conserved blocks with the D subgenome. These results suggest that *GAPDH* genes from different ancestral origins underwent differential fragmentation and loss, leading to asymmetric evolutionary patterns across subgenomes.

#### Cross-species collinearity analysis reveals the conservation and diversification of the wheat GAPDH gene Family

To examine evolutionary conservation, we performed collinearity analyses between wheat and other plant species, including monocots (*Oryza sativa* and *Zea mays*) and the dicotyledon *Arabidopsis thaliana*. Strong collinearity was observed between *Triticum aestivum* and both *Oryza sativa* and *Zea mays*, with 27 orthologous gene pairs identified in each comparison. These conserved regions were primarily localized on wheat chromosomes 2, 4, 6, and 7, showing clear collinearity relationships with multiple chromosomes in *Oryza sativa* and *Zea mays*. In contrast, collinearity between wheat and *Arabidopsis thaliana* was weak, with only two orthologous pairs identified within short conserved segments (Fig. 2B, Fig. S1B and Table S3). These results suggested that the *GAPDH* family underwent lineage specific expansion following the divergence of the Poaceae, while the core domains remained conserved prior to the monocot dicot split. The high genomic structural similarity between wheat and other grasses reflects their close phylogenetic affinity. These conserved collinear blocks provide a reliable structural framework for ortholog identification and functional annotation (Table 1).

**Table 1:** Summary of functionally characterized genes in collinear pairs

Name	Subfamily	Gene ID	function	Reference
*TaGAPDH1/2/3*	GAPA	Os04t0459500-02	photosynthesis	[32]
*TaGAPDH4/5/6*	GAPB	Os03t0129300-01	photosynthesis	[32, 53]
*TaGAPDH4/5/6*	GAPB	Zm00001eb002270_T001	drought and salt stress tolerance	[54]
*TaGAPDH8/12*	GAPC	AT1G13440.1	Low phosphate, vacuolar protein trafficking and plant senescence	[55–57]
*TaGAPDH8/10/12*	GAPC	Os02t0601300-01	Nematode Infection	[58]
*TaGAPDH8/10/12*	GAPC	Os04t0486600-01	Control gene	[59]
*TaGAPDH13/15/18*	GAPC	Os08t0126300-01	Salt tolerance	[60]

#### Intraspecific collinearity analysis

To explore the expansion patterns and evolutionary constraints of the wheat *GAPDH* gene family in common wheat, intra-species collinearity analysis was performed on hexaploid wheat. A total of 27 collinear gene pairs were identified, demonstrating that the expansion of the wheat *GAPDH* gene family was predominantly driven by segmental duplication, while no tandem duplication events were detected (Fig. 2C). Ka/Ks analysis showed that all duplicated gene pairs had Ka/Ks values lower than 0.3, indicating that this gene family underwent strong purifying selection, which maintained conserved amino acid sequences and stable core biological functions during evolution (Table S4). Collinear genes were mainly distributed on the homologous chromosome groups 6 A, 6B, 6D, 7 A, 7B, and 7D. Subfamily comparison suggested that the GAPC and GAPCP subfamilies contained more members owing to frequent segmental duplication, whereas the GAPA and GAPB subfamilies displayed fewer duplication events, slower evolutionary rates and higher sequence conservation (Fig. 2C). Collectively, segmental duplication combined with purifying selection jointly shaped the evolutionary landscape of the wheat *GAPDH* gene family, and distinct evolutionary patterns among subfamilies provide a molecular foundation for wheat environmental adaptation.

### Characterization of the *TaGAPDH *gene family

**Fig. 3:**
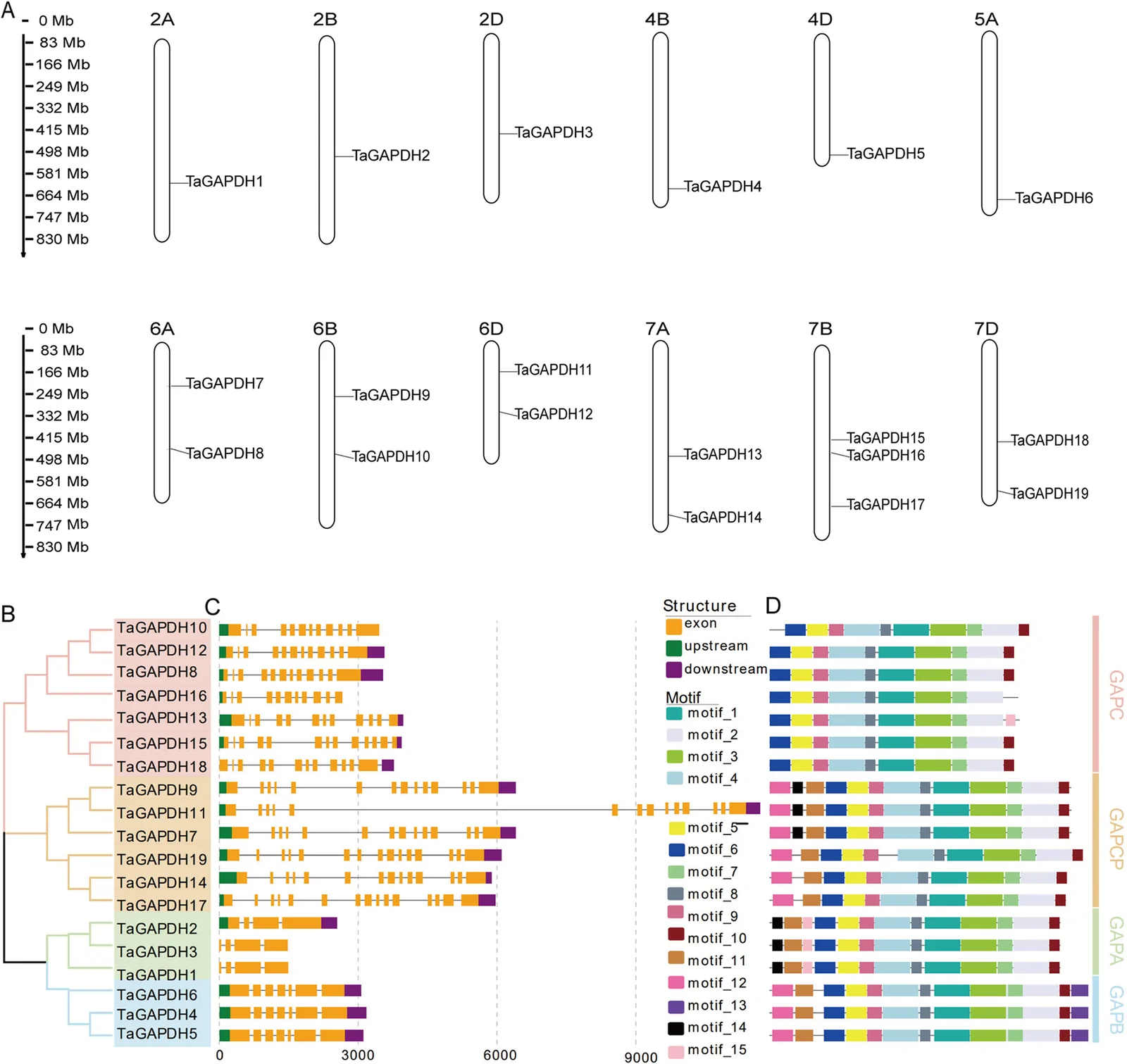
Chromosomal distribution, phylogenetic relationships, gene structures, and conserved motif composition of the wheat *GAPDH* gene family. **A** Chromosomal distribution of *TaGAPDH* genes. **B** Phylogenetic tree of TaGAPDH proteins constructed using their full-length amino acid sequences. Branch colors denote the different subfamilies. **C** Exon-intron organization of the identified *TaGAPDH* genes. Colored boxes represent exons, 5′untranslated regions (5′UTRs), and 3′untranslated regions (3′UTRs), whereas black lines represent introns. **D** Conserved motif composition of TaGAPDH proteins. Motif1-15 were represented by boxes of different colors

In this study, analysis of the chromosomal locations and gene structures of 19 *TaGAPDH* genes, as well as the physicochemical properties and conserved motifs of their encoded proteins. The amino acid length of TaGAPDH proteins ranged from 337 to 444 aa, with molecular weights varying between 36.53 and 46.93 kDa, reflecting relatively minor physicochemical variation within this gene family. Hydrophobicity and charge analyses further revealed obvious differentiation in protein properties, including 14 hydrophilic and five hydrophobic members, as well as six acidic, one neutral, and 12 basic proteins. Chromosomal localization analysis indicated an uneven distribution of *TaGAPDH* genes across 12 wheat chromosomes. Chromosome 7B harbored the largest gene number (3 members), whereas the remaining chromosomes contained only one or two genes (Fig. 3A). Multiple sequence alignment verified that all *TaGAPDH* proteins possessed the conserved Gp_dh_N and Gp_dh_C domains, suggesting highly conserved core structural characteristics (Fig. S2).

Based on phylogenetic clustering, *TaGAPDH* members were divided into four subfamilies (Fig. 3B), and gene structure analysis revealed distinct exon distribution patterns among these subfamilies (Table S6). The GAPCP subfamily displayed the most intricate gene structure with 14–15 exons, followed by the GAPC subfamily containing 11–13 exons. By contrast, the GAPB and GAPA subfamilies possessed relatively simple structures, with conserved exon numbers of seven and four, respectively. Members within the GAPB subfamily exhibited highly uniform gene structures and strong evolutionary conservation, implying stable and consistent biological functions (Fig. 3C and Tables S6). Conserved motif analysis showed that all TaGAPDH proteins contained Motifs 1 to 9, while Motif 10 was absent only in TaGAPDH13 and TaGAPDH16, reflecting high intrafamily sequence homology (Fig. 3D and Tables S7-S8) These conserved motifs are considered essential for maintaining protein spatial conformation and enzymatic activity. Additionally, several subfamily specific motifs were identified: Motif 11 was shared by GAPA, GAPB and GAPCP; Motif 12 was detected exclusively in GAPB and GAPCP; Motif 13 and Motif 15 were specifically enriched in GAPB and GAPA, respectively; and Motif 14 was present in partial members of GAPA and GAPCP. Collectively, these subfamily specific motifs may modify protein spatial structures, thereby driving functional divergence of *TaGAPDH* members during plant growth, development, and stress adaptation, and providing a critical molecular basis for the functional diversification of this gene family.

### Analysis of *cis*-regulatory elements in the promoters of the *TaGAPDH* gene family

**Fig. 4:**
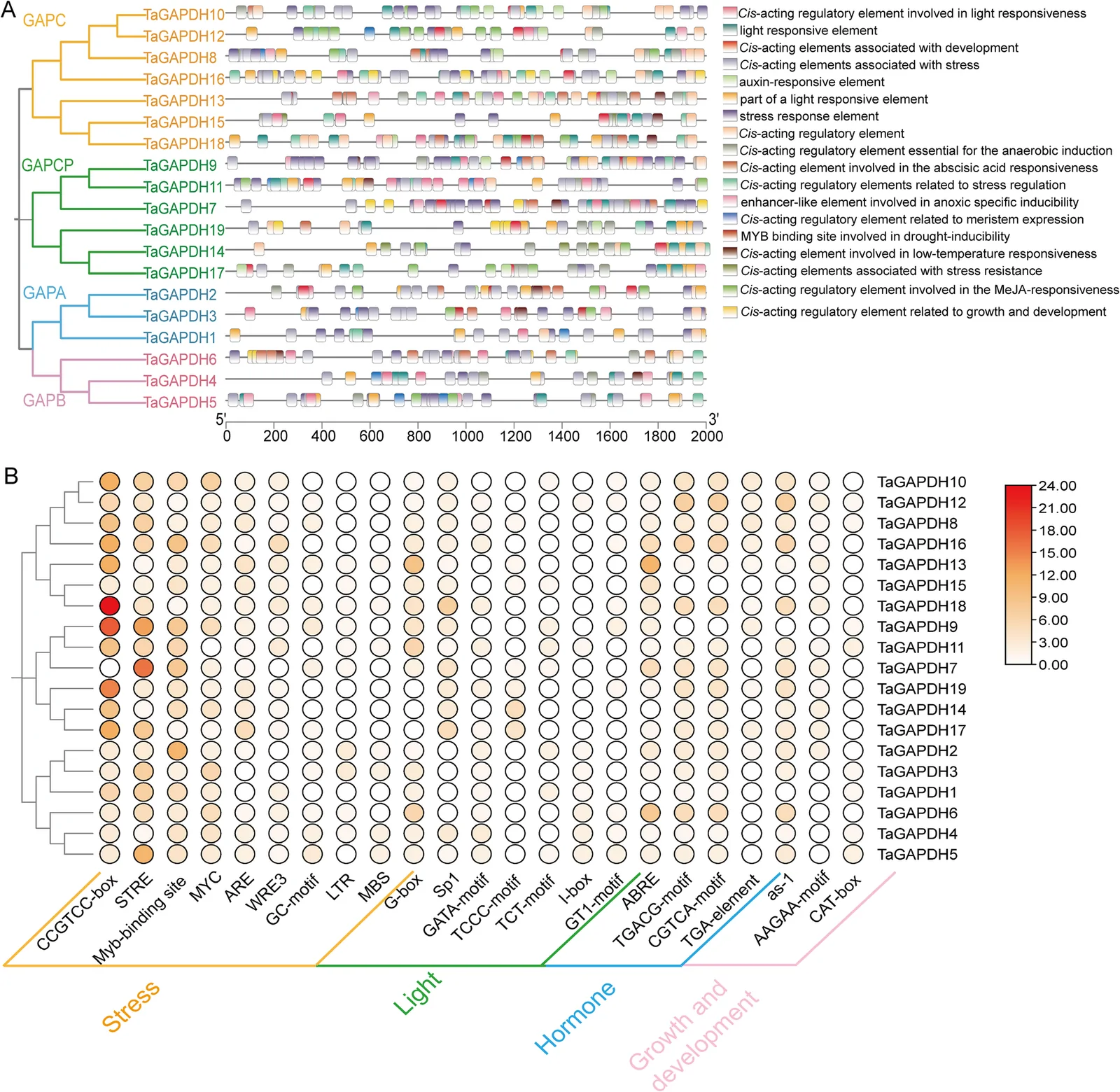
*Cis*-acting regulatory elements in *TaGAPDH* gene promoters. **A** Distribution of *cis*-acting elements within the 2000 bp upstream promoter regions of *TaGAPDH* genes. Grey horizontal lines represent the 2000 bp sequences relative to the translation start site; colored boxes indicate different types of *cis*-acting elements. **B** Heatmap showing the abundance of *cis*-acting elements in *TaGAPDH* gene promoters, with color intensity reflecting element frequency

To elucidate transcriptional regulatory mechanisms, 2000 bp promoter sequences upstream of the *TaGAPDH* start codons were analyzed. *Cis-*acting regulatory elements were identified and classified into four functional categories: stress responsiveness, light responsiveness, hormone responsiveness, and growth and development (Fig. 4A-B and Table S9). The enrichment of MYB, MYC, LTR, and MBS related *cis*-elements suggests that *TaGAPDH* genes may be integrated into complex transcriptional regulatory networks involved in responses to abiotic stresses, including drought, cold, and hypoxia. Notably, stress-responsive elements represented the most abundant category, among which the CCGTCC-box and STRE were the predominant motifs. Light-responsive elements, including G-box, Sp1, and GATA motifs, were the second most frequent, indicating light mediated transcriptional regulation. Additionally, the promoters contained numerous hormone related motifs (responsive to abscisic acid, methyl jasmonate, and auxin) and elements associated with meristem development. In summary, the *TaGAPDH* gene family appears to integrate environmental, hormonal, and developmental signals, playing dual roles in both stress adaptation and growth regulation.

### GO enrichment analysis of the *TaGAPDH* genes and protein-protein interaction network analysis of TaGAPDH proteins

**Fig. 5:**
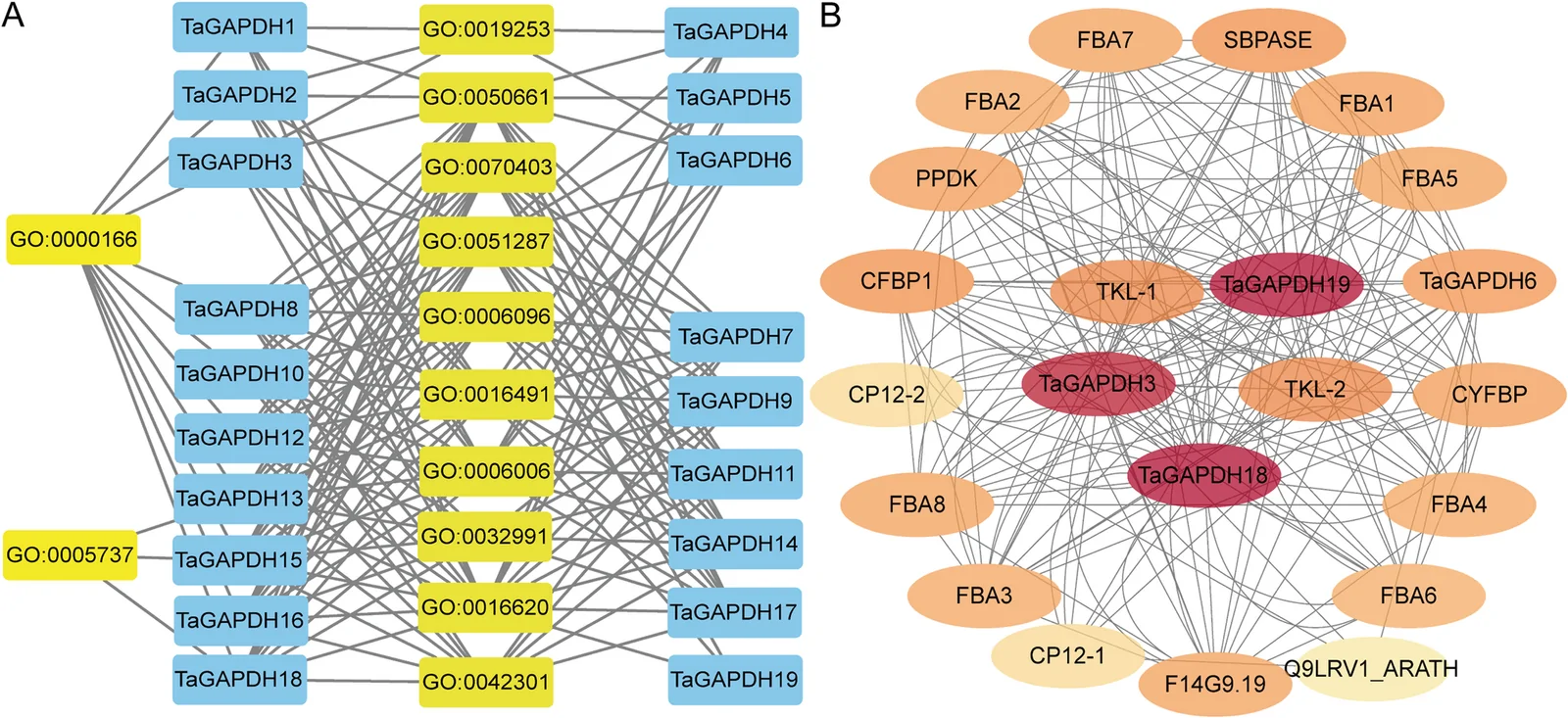
GO enrichment analysis of the *TaGAPDH* genes and protein-protein interaction network analysis of TaGAPDH proteins. **A** GO enrichment analysis. Yellow and blue blocks represent GO terms and *TaGAPDH* genes, respectively; grey lines indicate associations between them. **B** Predicted protein interaction network. Node color intensity reflects the connectivity and interaction strength, with darker nodes representing a higher number of interacting proteins

GO functional annotation was performed to elucidate the biological roles of *TaGAPDH* genes. Molecular function analysis showed enrichment in oxidoreductase activity and NAD/NADP binding, while biological process analysis highlighted involvement in glucose metabolism and the reductive pentose phosphate cycle (Fig. 5A and Table S10). These findings confirm that *TaGAPDH* genes function as NAD(P)-dependent oxidoreductases essential for photosynthetic carbon fixation and carbohydrate metabolism. To investigate protein-protein interactions, representative members from four subfamilies TaGAPDH3 (GAPA), TaGAPDH6 (GAPB), TaGAPDH18 (GAPC), and TaGAPDH19 (GAPCP) were selected as bait. Using the STRING database (*Arabidopsis thaliana* reference; confidence threshold ≥ 0.7) and Cytoscape visualization, 18 high confidence interacting proteins were identified, 15 of which interacted with all four baits (Fig. 5B and Table S11). These interactors were primarily enriched in the Calvin cycle, glycolysis, and carbon fixation pathways, including two CP12 proteins known to regulate GAPDH activity through complex assembly. The network also featured key enzymes such as CYFBP, which is involved in both carbon metabolism and ABA signaling. These results suggest that TaGAPDH proteins integrate carbon metabolism and hormone signaling through interactions with multiple functional proteins.

### Tissue-specific and developmental expression profiles of *TaGAPDH* genes

**Fig. 6:**
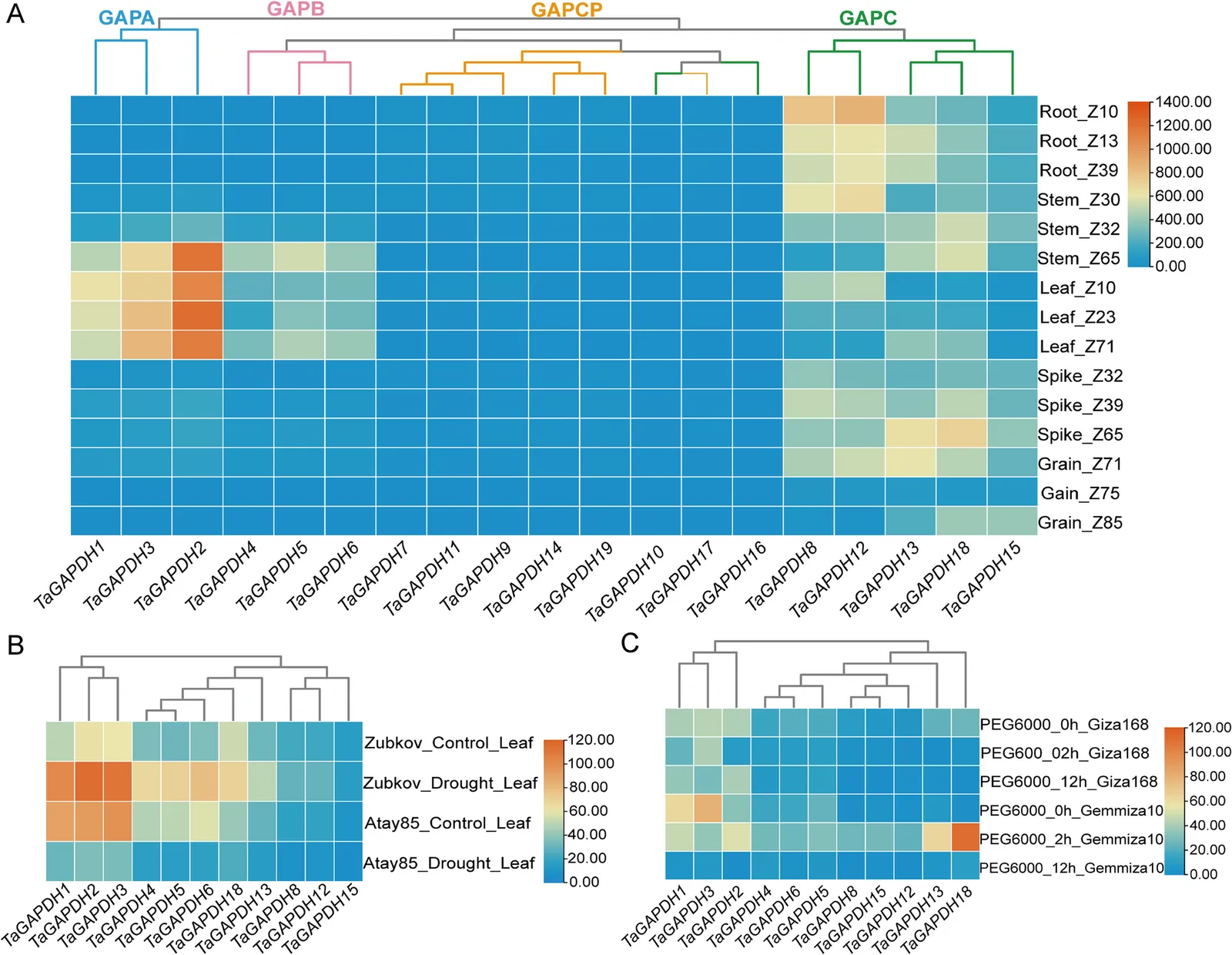
Spatiotemporal expression patterns and drought responses of *TaGAPDH* genes in wheat. **A** Expression profiles of *TaGAPDH* genes across different wheat tissues and developmental stages. The color scale indicates relative expression levels, ranging from low (light blue) to high (red). **B** Comparative expression patterns of 11 candidate *TaGAPDH* genes in drought-tolerant (Zubkov) and drought-sensitive (Atay85) wheat cultivars under drought stress. **C** Time-course expression profiles of the 11 candidate *TaGAPDH* genes in drought tolerant (Giza168) and drought-sensitive (Gemmiza10) wheat cultivars exposed to PEG 6000-induced osmotic stress. Samples were collected at 0, 2, and 12 h after treatment initiation

To investigate the biological roles of the wheat *GAPDH* gene family, expression profiles of 19 genes were analyzed across wheat roots, stems, leaves, spikes, and grains using public transcriptomic data (Fig. 6A and Table S12). Expression clustering was largely consistent with subfamily classifications, although two GAPC members (*TaGAPDH10* and *TaGAPDH16*) clustered within the GAPCP branch. The subfamilies exhibited distinct expression patterns, GAPC members (excluding the two divergent genes) showed high constitutive expression across all tissues (TPM > 26.41), suggesting they function as housekeeping genes to maintain basal metabolism and glycolytic homeostasis. Furthermore, expression profiling revealed that GAPA subfamily members were predominantly expressed in photosynthetic tissues (peaking in leaves, followed by stems and spikes) and early developing grains (Grain_Z71), but were barely detectable in roots and late developing seeds (Grain_Z85). This suggests a primary role in photosynthetic carbon assimilation and early energy accumulation. The GAPB subfamily exhibited a spatiotemporal expression pattern highly parallel to that of GAPA, albeit with lower overall transcript abundance, implying a synergistic regulation of energy metabolism in photosynthetic tissues. In contrast, the GAPCP subfamily maintained constitutively low expression across all examined tissues. In summary, *TaGAPDH* subfamilies have undergone clear functional specialization characterized by distinct evolutionary expression patterns. Based on tissue expression profiles and the presence of drought responsive promoter elements, 11 highly expressed genes from the GAPA, GAPB, and GAPC subfamilies (excluding *TaGAPDH10* and *TaGAPDH16*) were selected for drought response analysis.

In particular, transcriptome analysis revealed that all 11 *TaGAPDH* genes responded transcriptionally to drought stress, with members of the GAPA and GAPB subfamilies showing the most pronounced changes. Notably, these genes exhibited contrasting expression patterns between the two wheat cultivars: following drought treatment, their transcript levels increased by at least 1.84-fold in the drought-tolerant cultivar Zubkov, but decreased by at least 2.63-fold in the drought-susceptible cultivar Atay85 (Fig. 6B and Table S13). Similarly, under PEG6000-induced osmotic stress, the two wheat cultivars with contrasting drought tolerance displayed distinct expression patterns of *TaGAPDH* genes. After 2 h of PEG6000 treatment, most *TaGAPDH* genes showed only marginal expression changes in the drought-sensitive cultivar Giza168, with several genes even exhibiting reduced transcript abundance. In contrast, in the drought-tolerant cultivar Gemmiza, all *TaGAPDH* genes except *TaGAPDH1* and *TaGAPDH3* were upregulated (Fig. 6C and Table S14). Considering the expression clustering patterns and transcript abundances observed under both drought stress and PEG6000-induced osmotic stress, *TaGAPDH5* was selected as a candidate gene for subsequent functional validation of its potential role in drought tolerance.

### Construction of a transcriptional regulatory network for candidate *TaGAPDH* genes

**Fig. 7:**
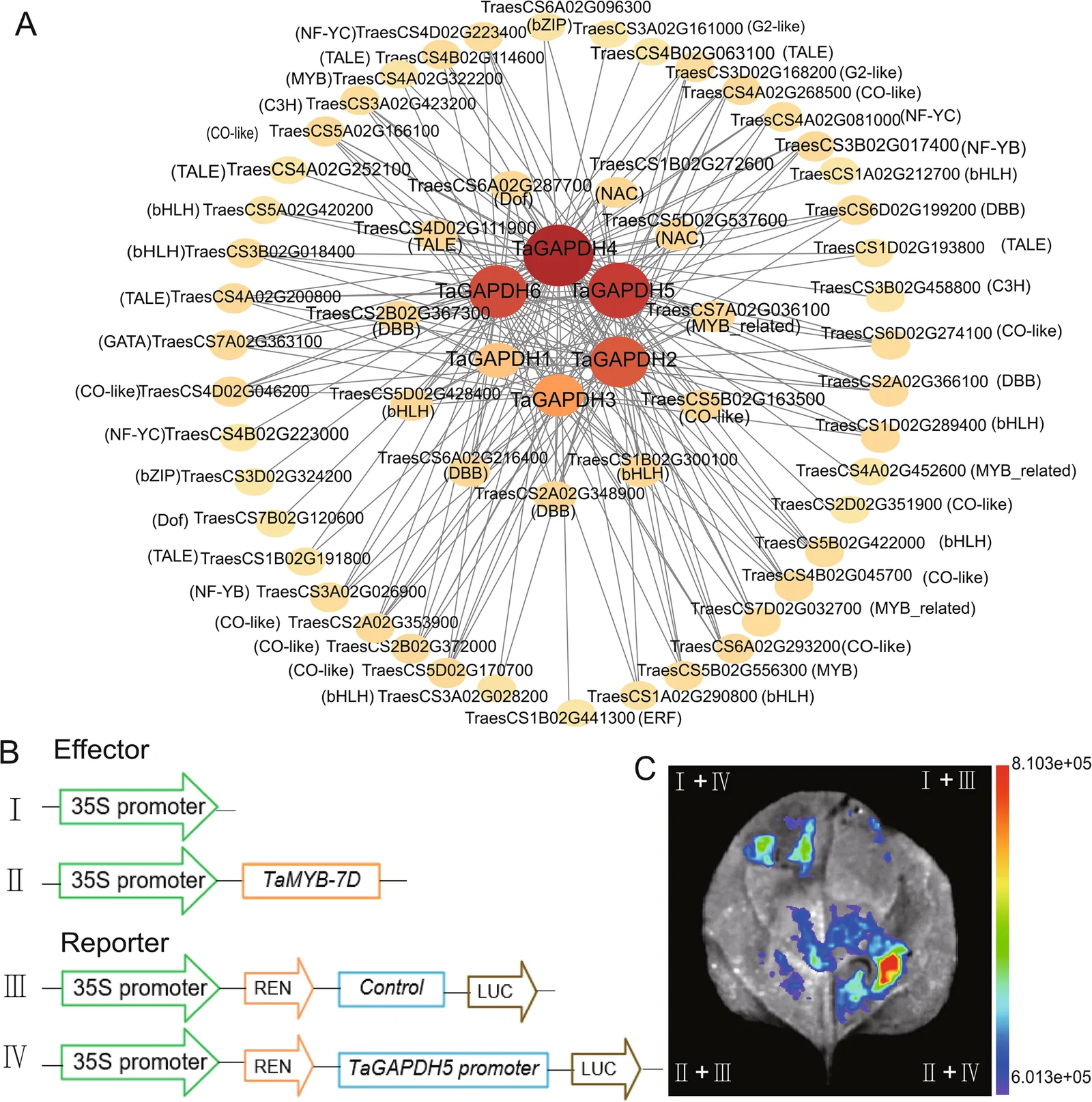
Expression-correlation network of six candidate *TaGAPDH* genes under drought stress and transcriptional activation of the *TaGAPDH5* promoter by TaMYB-7D. An expression-correlation network was constructed based on gene-expression profiles of wheat cultivars with contrasting drought tolerance under drought stress. Nodes represent individual genes, and node size is proportional to node degree. Edges denote significant positive Pearson’s correlations in expression between gene pairs (|*r*| > 0.95, *P* < 0.05). **A** Putative upstream regulatory network of *TaGAPDH* genes. **B** Schematic diagram of the dual-luciferase reporter assay used to evaluate the transcriptional regulatory activity of TaMYB-7D on the *TaGAPDH5* promoter. **C** Dual-luciferase reporter assays result

To preliminarily identify transcription factors potentially associated with the six drought-responsive *TaGAPDH* genes, candidate transcription factors were first predicted using the wheat Gene Regulatory Network (wGRN) platform, with thresholds of false discovery rate (FDR) ≤ 0.01 and enrichment fold ≥ 2 (Table S15). Subsequently, Pearson correlation coefficients were calculated between each *TaGAPDH* gene and the predicted candidate transcription factors using drought-related transcriptomic datasets. Gene TF pairs showing highly consistent expression trends were retained according to the criteria of ∣r∣≥0.95 and *P* < 0.05. Expression-trend correlation analysis showed that all 6 candidate *TaGAPDH* genes displayed significant and highly concordant expression patterns with different numbers of transcription factors, although substantial differences were observed in the numbers of associated transcription factors among individual *TaGAPDH* genes (Fig. 7A and Table S15-S16). *TaGAPDH1* was associated with the fewest transcription factors, with 13 transcription factors exhibiting highly correlated expression trends. This was followed by *TaGAPDH3* (23), *TaGAPDH2* (36), *TaGAPDH6* (39), and *TaGAPDH5* (43). By contrast, *TaGAPDH4* showed highly consistent expression trends with the largest number of transcription factors (52). These results suggest that individual *TaGAPDH* genes may exhibit distinct expression coordination patterns during drought responses and may be associated with different sets and numbers of transcription factors. In particular, the larger numbers of transcription factors showing highly concordant expression trends with *TaGAPDH4*, *TaGAPDH5*, and *TaGAPDH6* suggest that these genes may be involved in broader drought-responsive transcriptional processes. It should be noted that, given the limited sample size used for the correlation analysis, these results indicate expression-trend associations in the available drought-related transcriptomic datasets.

Based on expression profiling under drought and PEG6000-induced osmotic stress, analysis of coordinated expression trends among drought-responsive genes, and promoter *cis*-element prediction, we identified TaMYB-7D as a MYB-related transcription factor that may be involved in the transcriptional regulation of *TaGAPDH* genes. Across the expression datasets analyzed, the expression dynamics of *TaMYB-7D* showed similar trends to those of four *TaGAPDH* genes. Promoter sequence analysis further revealed the presence of putative MYB-recognition elements in the *TaGAPDH5* promoter. Because *TaGAPDH5* was strongly induced by both drought and PEG6000 treatments, we next assessed the potential effect of TaMYB-7D on *TaGAPDH5* promoter activity using a transient dual-luciferase reporter assay in *Nicotiana benthamiana* leaves. Compared with the empty effector control, co-expression of TaMYB-7D significantly increased luciferase activity driven by the *TaGAPDH5* promoter (Fig. 7B-C; Fig. S4). These results indicate that TaMYB-7D can enhance *TaGAPDH5* promoter activity in a heterologous transient expression system, suggesting that it may contribute to the transcriptional regulation of *TaGAPDH5 *gene under drought-responsive conditions.

### Structural prediction of the TaGAPDH5 protein and tissue-specific and drought-responsive expression analysis of the *TaGAPDH5* gene

**Fig. 8:**
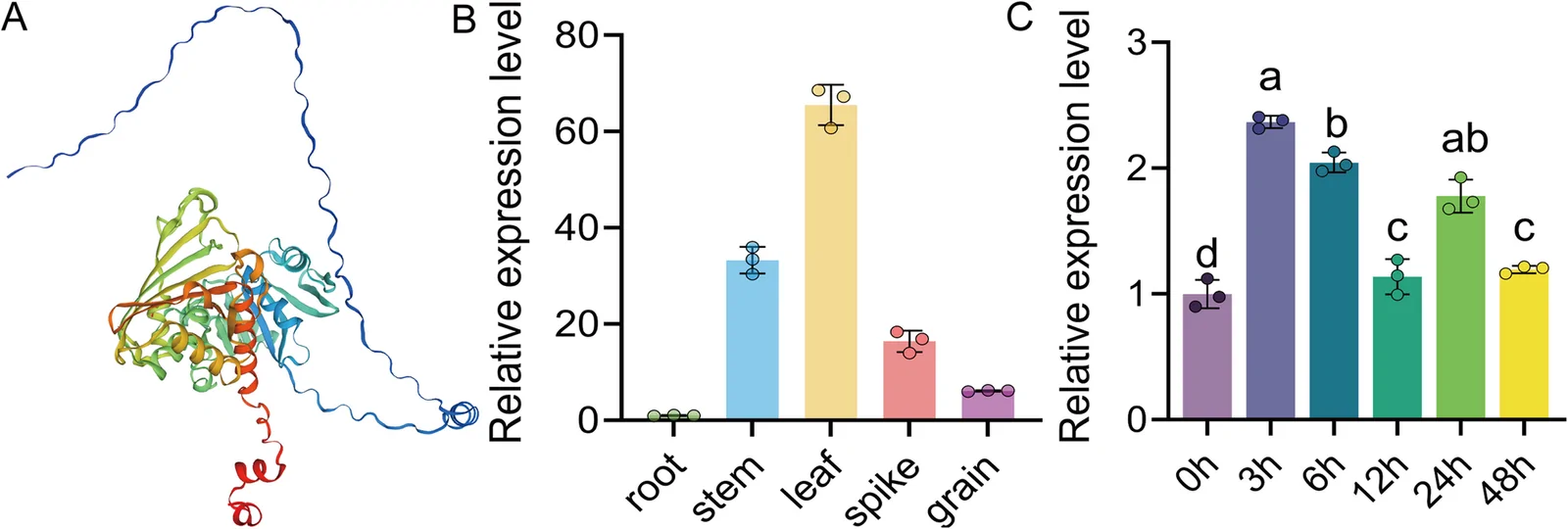
Structural prediction of the TaGAPDH5 protein and expression pattern analysis of the *TaGAPDH5* gene. **A** Homology based tertiary structure model of the TaGAPDH5 protein. **B** Tissue-specific expression profiles of *TaGAPDH5 *gene in different wheat tissues. **C** Relative transcript levels of *TaGAPDH5* gene at different time points following PEG-simulated drought treatment

To characterize the structural features of the TaGAPDH5 protein, its secondary and tertiary structures were predicted (Table S17). SOPMA analysis revealed that the secondary structure of TaGAPDH5 consists of 29.95% α-helices, 18.69% extended strands, and 51.35% random coils. The predominance of random coils provides high spatial flexibility and structural plasticity, facilitating potential substrate binding and protein-protein interactions. For tertiary structure homology modeling, the *Zea mays* ZmGAPDH protein (PDB: B4F8L7.1.A; LOC542290) served as the template. The resulting model exhibited a target-template sequence identity of 89.84% and a Global Model Quality Estimation (GMQE) score of 0.85 (Fig. 8A). These metrics indicate that the overall folding of the constructed model is highly reliable, making it suitable for subsequent functional site analysis and comparative structural studies.

RT-qPCR was performed to characterize the tissue-specific and drought-induced expression profiles of *TaGAPDH5*. Tissue expression analysis showed that *TaGAPDH5* was predominantly expressed in photosynthetically active leaves, whereas its transcript accumulation was extremely low in roots (Fig. 8B). Under PEG6000-simulated drought stress, the transcript abundance of *TaGAPDH5* displayed a transient induction pattern, increasing initially and subsequently declining. *TaGAPDH5* expression peaked at 3 h after PEG6000 treatment and then gradually decreased. These results indicated that *TaGAPDH5* expression was significantly induced by drought stress, suggesting a potential role for *TaGAPDH5 *gene in the drought-stress response of wheat (Fig. 8C).

### Heterologous overexpression of *TaGAPDH5* enhances drought tolerance in *Arabidopsis thaliana*

**Fig. 9:**
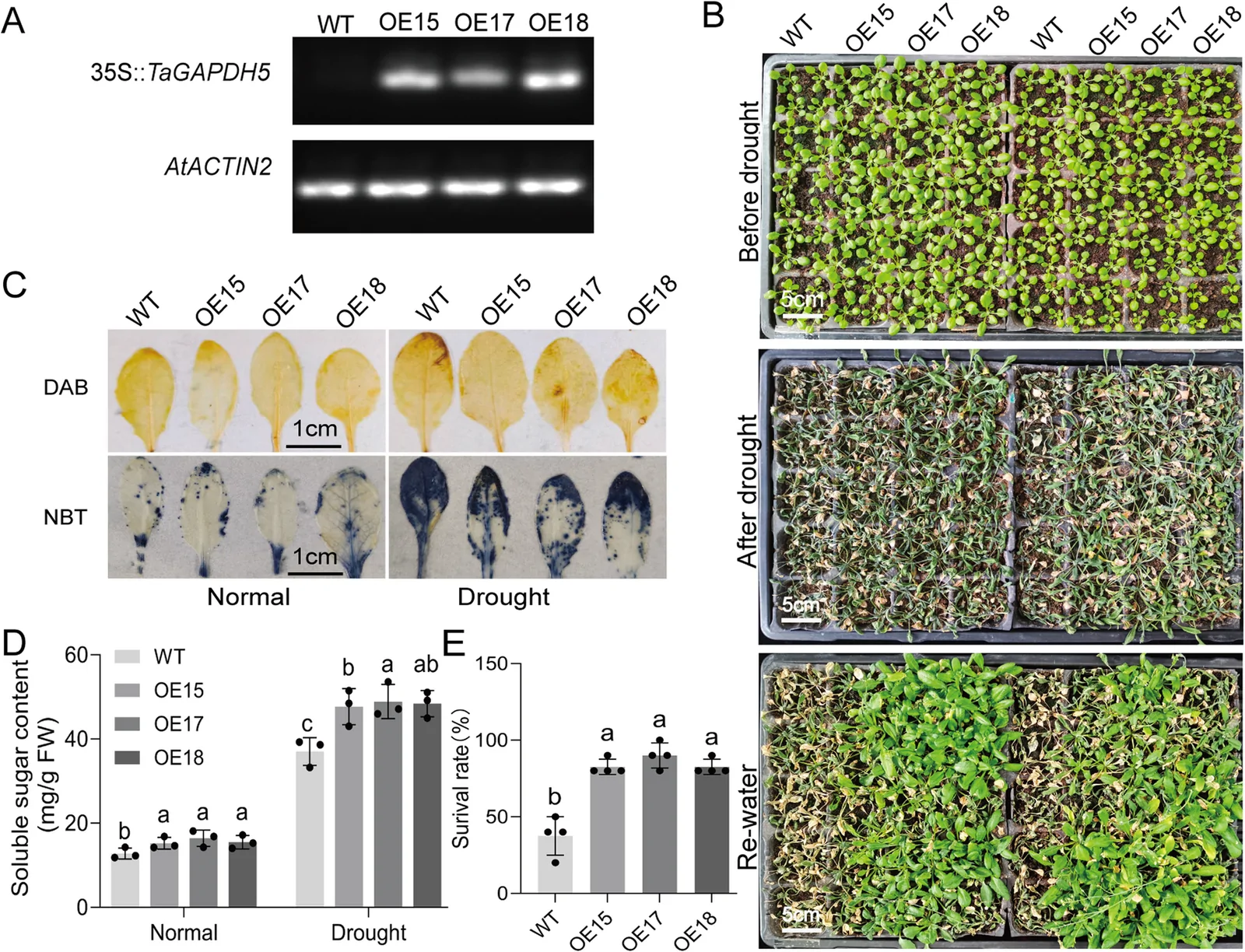
Drought stress phenotypes of WT and OE lines. **A** PCR‑based identification of WT and OE lines. WT, OE15, OE17 and OE18, independent overexpression lines. **B** Representative phenotypes of WT and OE seedlings under well-watered conditions, following 14 d of water-withholding drought stress, and after 3 d of rewatering. **C** DAB (top) and NBT (bottom) histochemical staining of leaves from WT and transgenic plants after 10 days of drought treatment, indicating accumulation of H₂O₂ and superoxide radicals, respectively. **D** Soluble sugar content in different plant lines under normal (well‑watered) and drought conditions (after 10 days of drought treatment). Data are presented as means ± SD (*n* = 3). Different letters indicate significant differences at *P* < 0.05 (Tukey’s test). **E** Survival rates of WT and OE plants after rewatering for 3 days following 14 days of drought stress. Values are means ± SD (*n* = 3 independent experiments, each with 10 plants per line), *P* < 0.05 (Tukey’s test)

To investigate the biological role of *TaGAPDH5* in plant responses to drought stress, *TaGAPDH5* overexpressing *Arabidopsis thaliana* lines were generated using the *Agrobacterium*-mediated floral dip method. Seeds harvested from transformed plants were screened on solid medium containing 100 mg L⁻¹ kanamycin, and kanamycin-resistant plants were subsequently verified by PCR. Three independent overexpression lines, OE15, OE17, and OE18, were selected for subsequent phenotypic analysis under drought stress (Fig. 9A).

Three-week-old WT and OE plants were subjected to drought stress. After 14 d of drought treatment, all OE lines exhibited less severe drought damage than WT plants, as evidenced by reduced wilting, greener leaves, and fewer senescent or dead leaves (Fig. 9B). To assess drought-induced ROS accumulation, leaves were collected after 10 d of drought stress and subjected to NBT and DAB histochemical staining. Compared with WT plants, OE lines exhibited weaker NBT and DAB staining, suggesting lower accumulation of superoxide anion (O2^−^ ) and hydrogen peroxide (H_2_O_2_ ), respectively (Fig. 9C and Fig. S5). In addition, soluble sugar content was measured after 10 d of drought stress (Fig. 9D and Fig. S5). Following 14 d of drought stress, plants were rewatered, and survival rates were assessed after 3 d of recovery. The OE lines showed a significantly higher survival rate than WT plants (Fig. 9B and E). Together, the *TaGAPDH5 *gene enhances drought tolerance in *Arabidopsis thaliana* overexpression lines and may represent a promising candidate for further functional validation in wheat.

## Discussion

### Asymmetric evolutionary patterns of the wheat *GAPDH* gene family during wheat polyploidization

The polyploidization is a primary driver of plant genome evolution and adaptive divergence, often involving genomic rearrangements and genetic introgression that provide extensive variation for crop improvement [61, 62]. Resulting from two allopolyploidization events, the complex genome of common wheat makes it an ideal model for studying polyploid evolution [63, 64]. The systematic exploration and effective utilization of beneficial alien genetic variation from wheat relatives represent an important strategy to alleviate the relatively narrow genetic base of modern bread wheat and enhance its breeding potential [65]. In this study, we analyzed the *GAPDH* genes across wheat species of different ploidy levels, revealing non uniform expansion and distinct subgenomic asymmetric evolution. While *GAPDH* genes in subgenome A expanded with increasing ploidy, those in subgenomes B and D remained stable, indicating a clear subgenomic bias in gene retention during wheat polyploidization.

The collinearity analysis across species and ploidy levels further elucidated these evolutionary mechanisms. While diploid and tetraploid wheats primarily carry single-copy homologs, hexaploid wheat displays a 1:3 copy distribution across subgenomes, suggesting that polyploidization induced gene dosage effects drove family expansion [66, 67]. Furthermore, evidence of fragment deletions and differential loss of ancestral homologs indicates that genomic structural rearrangements contributed to the asymmetric evolution of *TaGAPDH* genes [68, 69]. Cross-species comparisons showed that *TaGAPDH* genes share higher synteny and closer phylogenetic relationships with monocots (*Oryza sativa* and *Zea mays*) than with the dicot model *Arabidopsis thaliana*. Significant differences in conserved genomic segments between monocots and dicots suggest that the core GAPDH domain remained highly conserved during early plant evolution, followed by lineage specific expansion after the monocot dicot divergence.

Gene duplication analysis confirmed that segmental duplications primarily drove *TaGAPDH* genes expansion, with no evidence of tandem or retrotransposition mediated duplication. This pattern contrasts with *Populus deltoides*, which features tandemly duplicated *GAPDH* genes [70]. Natural selection likely promoted the differential retention of the wheat *GAPDH* gene family members across subgenomes, shaping a unique evolutionary lineage. This work provides a theoretical foundation for understanding the molecular mechanisms of gene retention and functional differentiation in polyploid wheat.

### Differential regulation of the wheat *GAPDH* gene family and the TaMYB-7D mediated drought response

Members of the wheat *GAPDH* gene family exhibit potentially divergent regulatory features. Promoter *cis*-element prediction and expression profiling revealed that *TaGAPDH* promoters are enriched in *cis*-regulatory elements associated with abiotic-stress and light responses, whereas their tissue-specific expression patterns are broadly consistent with those reported previously [21, 71]. Notably, members of both the GAPA and GAPB subfamilies showed pronounced responsiveness to drought treatment and PEG6000-simulated drought stress (Fig. 6B and C). Further expression-trend association analysis indicated that individual *TaGAPDH* genes may be regulated by distinct numbers of putative upstream transcription factors, predominantly from the NAC, MYB, bZIP, and G2-like families. These findings suggest that *TaGAPDH* genes may participate in a complex drought-responsive regulatory network coordinated by multiple transcription-factor families [14, 72–75]. In particular, the GAPB subfamily displayed more complex putative transcriptional regulatory patterns, implying a potentially important role in the wheat drought-response network. In addition, protein structural prediction indicated that TaGAPDH5 has substantial conformational plasticity (Table S17), a feature that may facilitate protein interactions required for metabolic regulation and signal transduction.

Dual-luciferase reporter assays indicated that TaMYB-7D enhanced *TaGAPDH5* gene promoter activity in a heterologous transient expression system. This observation is consistent with the predicted *cis*-regulatory architecture of the *TaGAPDH5* gene promoter, which contains multiple putative MYB and MYC binding sites. However, the dual-luciferase reporter assay only demonstrated that TaMYB-7D modulates the activity of the *TaGAPDH5* promoter and did not establish direct binding between TaMYB-7D and the promoter. Therefore, whether TaMYB-7D directly binds to the *TaGAPDH5* promoter requires further validation using complementary approaches, such as yeast one-hybrid assays, electrophoretic mobility shift assays (EMSAs), and chromatin immunoprecipitation followed by quantitative PCR (ChIP-qPCR). MYB-related transcription factors have been implicated in diverse drought-adaptive processes; for example, *MaMYBR30* and *SlMYB1L* positively regulate drought tolerance through ROS homeostasis and ABA signaling, whereas *TrMYB130* negatively regulates drought tolerance [76–78]. Collectively, our findings suggest transcriptional diversification within the wheat *GAPDH* family and support a putative regulatory relationship between the TaMYB-7D transcription factor and *TaGAPDH5*, which may influence glycolysis-related processes during drought responses.

### *TaGAPDH5* as a potential candidate gene for drought tolerance in wheat

Identifying genes associated with drought tolerance is essential for elucidating the molecular basis of stress adaptation in wheat and for developing drought-resilient cultivars. Increasing evidence suggests that GAPB subfamily members may participate in plant responses to diverse environmental stresses. Phylogenetic analysis placed *TaGAPDH5* within the GAPB subfamily and showed that it is closely related to *Oryza sativa OsGAPB* and *Zea mays ZmGAPB*. In *Oryza sativa*, *OsGAPB* contributes to adaptation to low-light conditions by modulating chlorophyll biosynthesis and photosynthetic efficiency [53], whereas loss of function mutations *ZmGAPB* increase sensitivity to drought and salinity and are associated with enhanced oxidative damage, impaired photosynthetic performance, and reduced antioxidant capacity [54]. Similarly, *ThGAPB* has been reported to protect the photosynthetic apparatus and promote plant survival under adverse conditions, potentially by sustaining cellular energy metabolism and limiting reactive oxygen species (ROS) accumulation [79]. These findings prompted us to investigate whether *TaGAPDH5* might also be involved in drought stress responses. In this study, drought strongly induced *TaGAPDH5* gene expression. Heterologous overexpression partially alleviated drought-induced growth inhibition, reduced ROS accumulation, and improved plant performance (Figs. 8C and 9C). OE lines accumulated more soluble sugars than WT plants, particularly under drought (Fig. 9D). Given the reported roles of GAPB proteins in photoprotection and stress-related energy metabolism, increased sugar accumulation may facilitate osmotic adjustment and energy supply. However, measurements of antioxidant enzyme activities, malondialdehyde, relative water content, osmotic potential, and GAPDH activity are needed to clarify the underlying mechanisms. These findings suggest a possible role for *TaGAPDH5* gene in drought responses and support its further functional validation and evaluation for wheat drought tolerance improvement.

## Conclusions

In this study, 5 and 6 GAPDH family members were identified in two diploid wheat progenitors, respectively, whereas 13 and 19 members were identified in tetraploid and hexaploid wheat, respectively. Integrative analyses of phylogeny, synteny, gene structure, *cis*-regulatory elements, and expression networks systematically characterized the evolutionary dynamics and regulatory divergence of the wheat *GAPDH* gene family, as well as its potential involvement in drought stress responses. The main conclusions are as follows:

1. The wheat *GAPDH* gene family underwent asymmetric expansion during wheat polyploidization, primarily driven by segmental duplication. Differential gene retention among subgenomes may be associated with gene dosage balance.
2. Differences in gene structure and expression patterns among *TaGAPDH* gene family members suggest potential regulatory and functional divergence. TaMYB-7D may regulate *TaGAPDH5* gene promoter activity, suggesting a putative regulatory association involved in the drought response.
3. Heterologous overexpression of *TaGAPDH5* gene alleviated drought-induced growth inhibition and enhanced drought tolerance in *Arabidopsis thaliana*, indicating its potential utility for improving drought tolerance in wheat.

Collectively, this study elucidates the evolutionary and regulatory features of the wheat *GAPDH* gene family. As the current functional evidence is derived mainly from bioinformatic analyses and a heterologous system, future genetic, biochemical, and field based studies in wheat are required to establish the endogenous function and breeding value of this regulatory module.

## Supplementary Information

Supplementary Material 1. Supplemental Figures. Fig. S1 Synteny analysis of *GAPDH* genes among different species. Fig. S2 Multiple sequence alignment of TaGAPDH proteins. Fig. S3 Classification and abundance of *cis*-regulatory elements in the promoter regions of *TaGAPDH* genes. Fig. S4 Quantitative analysis of luciferase activity. Fig. S5 Representative phenotypes of WT and OE *Arabidopsis thaliana* plants after 10 d of drought stress.

Supplementary Material 2. Supplemental Data. Tables S1-S17. The primer sequences used in this study and Gene related information.

## Data Availability

All data generated and analyzed in this study are included within the article and its Supplementary Information. Raw datasets that support the findings of this study are available from the corresponding author upon reasonable request.

## Abbreviations

GAPDH: Glyceraldehyde-3-phosphate dehydrogenase
PRODH: Proline dehydrogenase
SOD: Superoxide dismutase
POD: Peroxidase
CAT: Catalase
ROS: Reactive oxygen species
GAPA/B/C: Glyceraldehyde-3-phosphate dehydrogenase A/B/C subunit
NBT: Nitroblue Tetrazolium
DAB: 3,3′-Diaminobenzidine
