# Supplementary material for: Evolutionary characteristics of the GAPDH gene family and functional characterization of TaGAPDH5 in wheat
Source: BMC Plant Biol. 2026 Sep 24;26:1696. doi: 10.1186/s12870-026-09787-x (PMC13637277; doi:10.1186/s12870-026-09787-x)
Supplement: Supplementary file 1 [file 12870_2026_9787_MOESM1_ESM.docx]

**Additional files**

**Fig. S1** Interspecific collinearity analysis. (A) Collinearity between *Triticum aestivum*, and *Triticum urartu*. (B) The collinearity between *Triticum aestivum* and *Zea mays*.

**Fig. S2** Multiple sequence alignment of TaGAPDH protein sequences.

**Fig. S3** Abundance and classification of *cis*-acting elements. Colored bars represent the total count of elements within each major functional category.

**Fig. S4** Quantitative analysis of luciferase activity. The LUC/REN ratio was calculated to evaluate the transcriptional activation activity.

**Fig. S5** Representative phenotypes of WT and OE *Arabidopsis thaliana* plants after 10 d of drought stress.
